# Endothelial failure and rejection in recipients of corneas from the same donor

**DOI:** 10.1136/bmjophth-2021-000965

**Published:** 2022-08-17

**Authors:** Lewis Downward, Mahmoud Ahmed, Cathy Hopkinson, Vito Romano, Elinor Curnow, Stephen B Kaye

**Affiliations:** 1 Statistics, NHS Blood and Transplant Organ Donation and Transplantation Directorate, Bristol, UK; 2 Ophthalmology, Royal Liverpool University Hospital, Liverpool, UK; 3 Department of Eye and Vision Science, University of Liverpool, Liverpool, UK

**Keywords:** Cornea, Eye (Tissue) Banking

## Abstract

**Objective:**

To determine whether patients who receive corneas from the same donor have similar risks of endothelial failure and rejection.

**Methods and Analysis:**

Patients with Fuchs endothelial dystrophy (FED) and pseudophakic bullous keratopathy (PBK) who received their first corneal transplant between 1999 and 2016 were analysed. Patients receiving corneas from donors who donated both corneas for the same indication were defined as ‘paired’. Gray’s test was used to compare the cumulative incidence of endothelial failure and rejection within 5 years post-transplant for ‘paired’ and ‘unpaired’ groups. Cox regression models were fitted to determine whether there was an association between recorded donor characteristics (endothelial cell density (ECD), age and sex and endothelial graft failure and rejection.

**Results:**

10 838 patients were analysed of whom 1536 (14%) were paired. The unpaired group comprised 1837 (69%) recipients of single corneal donors and 7465 (69%) donors who donated both corneas for another indication. ECD was lower for unpaired single cornea donors (p<0.01). There was no significant difference in endothelial graft failure or rejection between paired and unpaired groups for FED (p=0.37, p=0.99) or PBK (p=0.88, p=0.28) nor for donor ECD, age, sex and paired donation after adjusting for transplant factors (across all models p>0.16 for ECD, p>0.32 for donor age, p>0.14 for sex match and p>0.17 for the donor effect).

**Conclusion:**

The absence of a significant difference in graft outcome for corneal transplants for FED and PBK between paired and unpaired donors may reflect a homogeneous donor pool in the UK.

**What is already known on this topic?**

- Pairs of donor eyes from the same donor have similar endothelial cell densities and is associated with similar post corneal transplant central corneal thickness.

**What this study adds?**

- We found similar endothelial graft rejection and failure in recipients of corneas from paired and unpaired donors.

**How this study might affect research, practice or policy?**

- The borderline significant donor effect in Fuchs endothelial dystrophy patients for endothelial graft survival would suggest that there may be donor factors which have not been identified. This requires investigation.

## Introduction

The cornea differs from other tissues or organs in that in its healthy state it is a relatively privileged site for transplantation due to the absence of blood and lymphatic vessels (except for the marginal corneal arcades)^1^ and a relative paucity of mature antigen presenting cells.^2^ Despite this, corneal graft failure remains significant with an overall 5-year graft survival for Fuchs endothelial dystrophy (FED) and pseudophakic bullous keratopathy (PBK) of 79% (95% CI 76% to 81%), and 59% (95% CI 55% to 63%), respectively.^3 4^ Corneal transplants fail predominantly from endothelial failure as these cells do not divide and depend on survival of the donor endothelium. Corneal graft rejection and/or inflammation in the recipient are significant causes of endothelial graft failure.^3 5^

Both donor and recipient factors play a role in graft rejection and failure. Many of the recipient risk factors associated with rejection are well recognised, including young recipient age, number of previous transplants, vascularisation, previous rejection episodes and the indication and type of transplant.^6 7^ Similarly, donor factors such as endothelial cell density (ECD), donor age, H-Y incompatibility have been shown to play variable roles in graft rejection and failure. Many donor factors such as race,^8^ lens status,^9^ harvesting^10–12^ and preservation techniques^8 9 11 13–19^ have been studied, but the evidence is often conflicting. A comprehensive summary of the donor factors that have been investigated to influence graft outcome and the associated evidence is provided in online supplemental table 1.^8–68^

10.1136/bmjophth-2021-000965.supp1 Supplementary data

Brightbill and Kaufman drew attention to investigating the outcomes of penetrating keratoplasty (PK) between recipients of paired donor eyes.^69^ This was based on the findings that pairs of donor eyes have minimal differences in ECD.^70^ They found that despite a range of recipient corneal diseases, paired grafted corneas showed remarkable similarity in postoperative central corneal thickness.

In this study, the demographics and endothelial outcomes for ‘paired’ corneal transplants were compared with ‘unpaired’ single corneal donor transplants and from donors who donated both corneas but for an alternative indication or regraft. An association between cause of failure and type of rejection for each patient of paired donor transplants was also investigated. The potential similarity in outcomes between transplant recipients of the same donor as well as potential differences between single corneal donors was of interest. It is unclear if corneas from donors where only one eye is suitable for transplant, differ in quality from corneas from donors where both eyes are issued for transplantation and whether this has a bearing on graft outcome. Patients with FED and PBK patients were selected because rejection and failure is due to endothelial failure and in particular, patients with FED are a relatively homogeneous group with few risk factors. Additionally, other donor factors were explored such as donor age, ECD and gender mismatch to determine whether these factors were associated with an increased hazard of endothelial graft failure and rejection. As well as recorded donor factors, an unknown donor effect was fitted to account for unexplained donor to donor variation and for any correlation between pairs of corneas from the same donor.

## Methods

Data were obtained from the UK Transplant Registry managed by NHS Blood & Transplant (NHSBT). NHSBT collect data on all corneal transplants performed in the UK. Completion of a transplant surgery record and follow-up forms at 1, 2 and 5 years post-transplant is required for all surgeons undertaking corneal transplantation in the UK.

### Patients

Data from all adult patients (aged 16 or above) with an indication of FED or PBK who received their first corneal transplant using NHSBT supplied corneas from adult (aged 16 or above) donors, between 1 April 1999 and 31 March 2016 in the UK were included. The cohort was also restricted to patients who received a PK or endothelial keratoplasty (EK) with an ECD greater than 2200 cells/mm^2^.

Only patients receiving corneas from donors who had donated both corneas for a first transplant for the same ocular disease were defined as ‘paired’ transplants (group A) in the analysis. ‘Unpaired’ transplants were defined as patients receiving a cornea from donors who had donated a single cornea (group B, unpaired single cornea donor cohort) as well as patients receiving a cornea from donors who had donated two corneas where one cornea was transplanted for a re-graft or for another ocular disease (group C, unpaired double cornea donor cohort).

### Primary outcome measures

Endothelial graft failure: graft failure due to endothelial decompensation, primary graft failure or recurrence of the original disease. In addition to patients who had not experienced graft failure by the last follow-up, patients who experienced graft failure from any non-endothelial cause were censored at the time of graft failure.

Endothelial rejection: The first episode of endothelial rejection. In addition to patients who had not experienced rejection by the last follow-up, patients who experienced rejection from any non-endothelial cause were censored at the time of rejection.

### Statistical analysis

Demographic data were reported as numbers, percentages and were compared using Fisher’s exact test. The causes of failure or rejection were compared for each pair of transplanted corneas for both FED and PBK combined. The cumulative incidence of endothelial graft failure and rejection were described for the paired and unpaired groups using the Aalen-Johansen estimator^71^ and compared using a Gray’s test.^72^ Graft failure and rejection from other causes were the competing events.

Cause-specific Cox regression analysis was used to determine whether there was an association between donor factors and endothelial graft failure and endothelial rejection within 5 years post-transplant, by indication for transplant. The effect of unknown donor factors was accounted for by including a random donor effect in each model. This also allowed for correlation between outcomes for two recipients of corneas from the same donor. A sensitivity analysis was performed to validate the results by repeating the same analysis using both paired and unpaired transplants. The donor characteristics of ECD, donor age and donor-recipient sex match were included in each model. Other donor morbidity such as diabetes, or medications were not available. The recipient and transplant characteristics considered for inclusion in each model were: recipient age, sex and high risk recipient (vs low risk; high risk defined as any ocular surface disease or corneal vascularisation at time of transplant), graft type, risk of glaucoma at the time of transplant, as well as post-operative factors: cataract surgery, elective removal of all sutures, selective suture adjustment or removal, loose or broken suture, glaucoma medication, other immunosuppressants and wound leak complications. Postoperative risk factors were included as time dependent variables.

In all models, for each recipient and transplant characteristic considered for inclusion, a value of p≤0.1 based on the likelihood ratio test (comparing models with and without the risk factor) was deemed sufficient evidence for the risk factor to be included in each given model. If there were <15% of data missing, a complete-case analysis was used for modelling.

### Patient and public involvement

Patients and public were not involved in the design, conduct, analysis or writing of the manuscript

## Results

Data from 11 381 patients were included who had undergone a first corneal transplant for FED or PBK and met all the cohort criteria during the period of interest. A total of 543 (5%) were excluded from the analysis due to a lack of follow-up data. Of the 10 838 patients with follow-up data, 1536 (14%) patients received a cornea from a donor who had donated both corneas to two patients with the same indication for transplantation (group A paired). The unpaired transplants consisted of 9302 (86%) patients who received corneas either from single cornea donors (group B 1837, 17%) or from donors who donated one cornea for FED or PBK patients and the other cornea was used for either a regraft or for another ocular disease (group C 7465, 69%). Characteristics at the time of transplant for paired and unpaired transplants are compared in table 1.

**Table 1:** Characteristic comparisons of the paired and unpaired cohorts

	FED							PBK						
	Paired (group A)		Unpaired				P value	Paired (group A)		Unpaired				P value
			B. single		C. double					B. single		C. double		
	N	%	N	%	N	%		N	%	N	%	N	%	
Donor age group*	1	0					<0.01	2	1					<0.01
16–40			6	1	46	1				6	1	51	2	
41–60	52	11	105	10	621	15		19	7	77	9	456	14	
61–75	232	48	440	44	1918	46		109	39	307	37	1351	41	
>75 years	203	42	460	45	1567	38		150	54	436	53	1455	44	
Endothelial cell density†							<0.01							<0.01
2200–2700	681	70	784	78	2933	71		386	69	663	80	2347	71	
>2700	295	30	227	22	1219	29		174	31	163	20	966	29	
Donor–recipient sex match							0.04							0.40
Male–female	341	35	336	33	1546	37		171	31	277	34	1038	31	
Other combinations	635	65	675	67	2606	63		389	69	549	66	2275	69	
Graft type	414	42					0.17	322	58					0.64
PK			467	46	1889	45				492	60	1917	58	
EK	562	58	544	54	2263	55		238	43	334	40	1396	42	
Risk of glaucoma	922	94					0.24	434	78					0.93
No			957	95	3967	96				647	78	2587	78	
Yes	54	6	54	5	185	4		126	23	179	22	726	22	
Total Transplants	976	100	1011	100	4152	100		560	100	826	100	3313	100	

*Per donor rather than by transplant.

†When comparing ECD as a continuous variable we found that the difference in ECD for the cohorts was still significant for both FED and PBK (p<0.001)

ECD, endothelial cell density; EK, endothelial keratoplasty; FED, Fuchs endothelial dystrophy; PBK, pseudophakic bullous keratopathy; PK, penetrating keratoplasty.

For many of the characteristics, there was some evidence of a statistical difference between the cohorts. Donors were younger in the unpaired double cornea donor cohort for PBK (p<0.01) and for FED (p<0.01) compared with the paired, and unpaired single cornea donor cohorts. ECD was lower for the unpaired single cornea donor cohort for PBK (p<0.01) and FED (p<0.01). The unpaired double cornea donor cohort had a slightly higher proportion of male to female transplants for FED (p=0.04) compared with the other two cohorts. Overall, however, the paired and unpaired cohorts were of sufficient similarity to conclude that the paired cohort was representative of the entire first corneal transplant cohort.

### Causes of failure and rejection for pairs of donor corneas

In cases where both recipients experienced graft failure, endothelial decompensation was the most common cause of failure (N=7) and endothelial rejection was the most common cause of rejection (N=4). Across all causes of failure and rejection, the numbers of cases were very small. For this reason, a formal statistical test comparing causes of failure and rejection was not performed. Overall, the results indicated that there was not a relationship between the causes of graft failure or the causes of graft rejection between paired cornea donor recipients. There were 56 primary graft failures in the paired cohort (online supplemental table 2), 17 were in recipients of a PK, and the remaining 39 were for EK. Of the 56 primary graft failures, 25 were in FED patients (4 PK, 21 EK) and 31 were in PBK (13 PK, 18 EK).

10.1136/bmjophth-2021-000965.supp2 Supplementary data

### Endothelial failure and rejection

**Figure 1:**
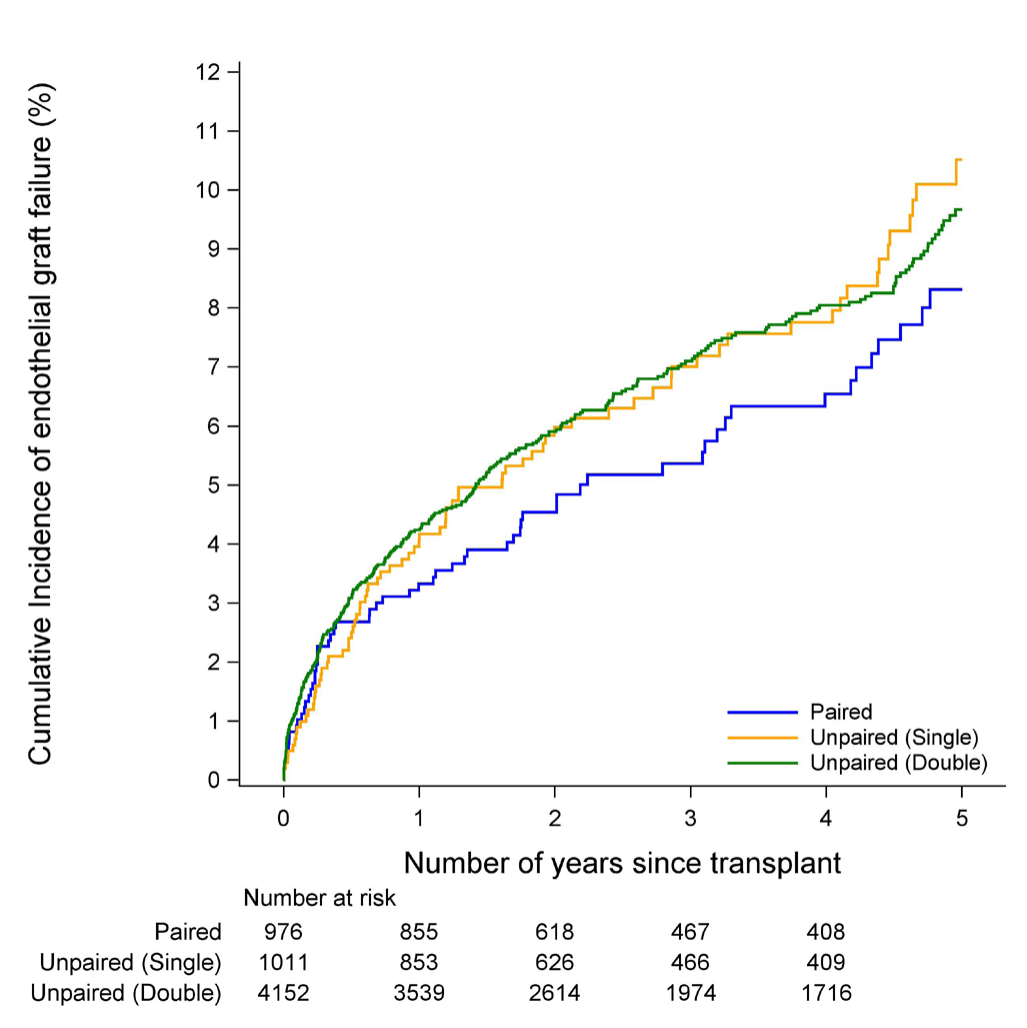
Cumulative incidence of endothelial graft failure in fed recipients.

**Figure 2:**
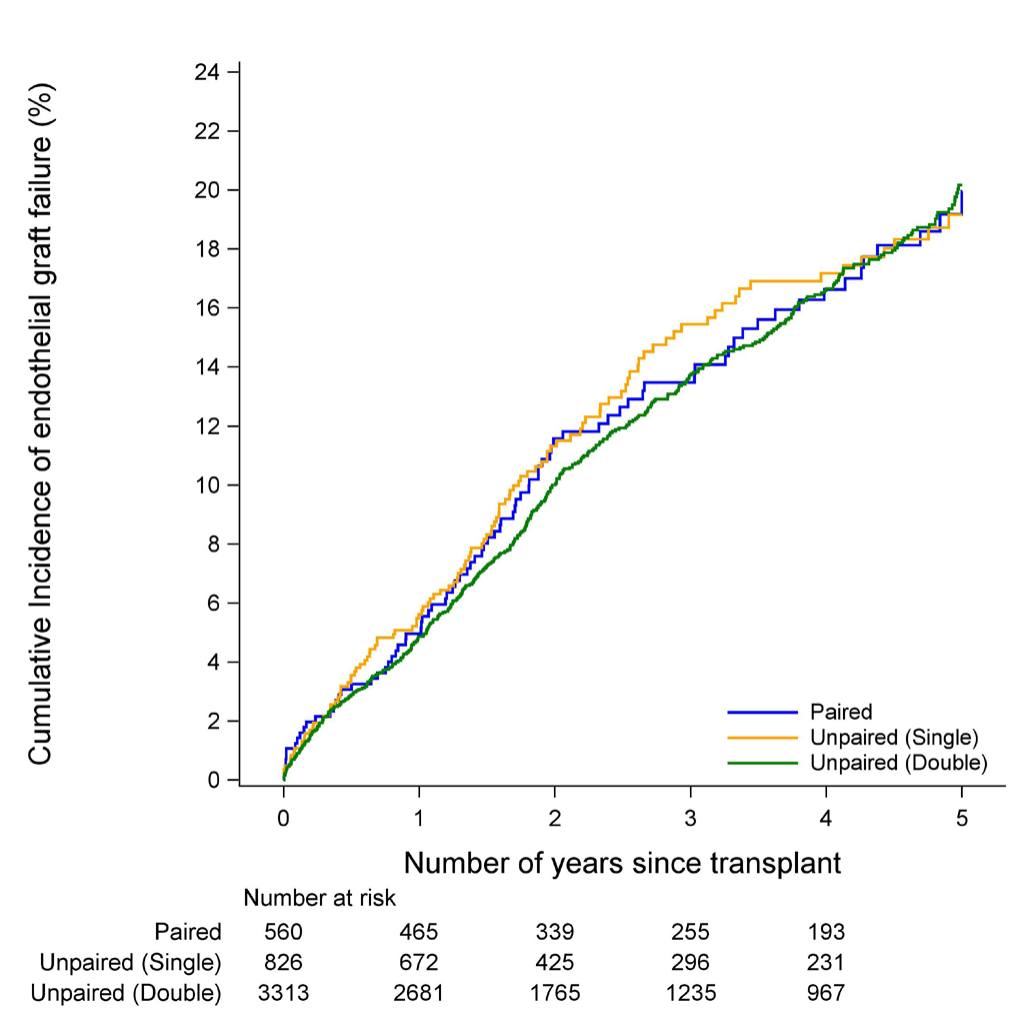
Cumulative incidence of endothelial graft failure in PBK recipients. PBK, pseudophakic bullous keratopathy.

**Figure 3:**
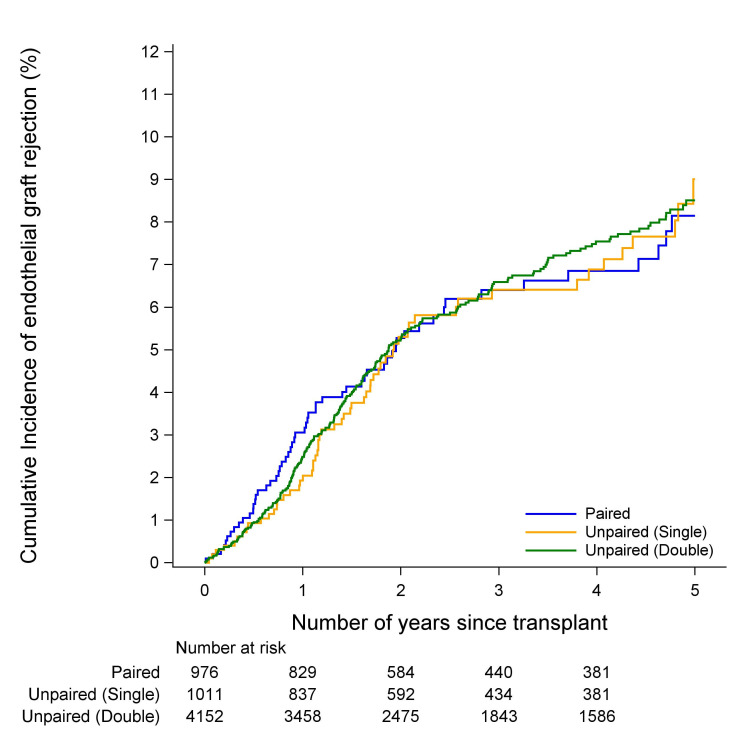
Cumulative incidence of endothelial graft rejection in fed recipients.

**Figure 4:**
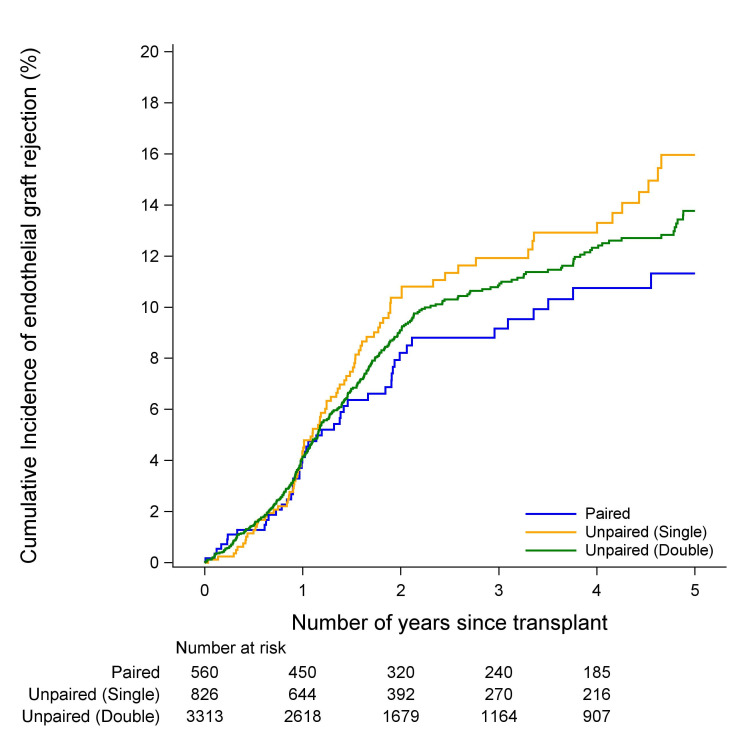
Cumulative incidence of endothelial graft rejection in PBK recipients. PBK, pseudophakic bullous keratopathy.

There was no evidence of a difference in the incidence of endothelial graft failure between paired, unpaired single cornea donor or unpaired double cornea donor cohorts in either FED or PBK (p=0.37 and p=0.88, figures 1 and 2, respectively). Similarly, there was no evidence of a difference in the incidence of endothelial graft rejection between the paired, unpaired single cornea donor or unpaired double cornea donor cohorts in either FED or PBK (p=0.99 and p=0.28, figures 3 and 4, respectively). The types of rejection are included in online supplemental table 3.

10.1136/bmjophth-2021-000965.supp3 Supplementary data

In PBK grafts, the endothelial graft rejection rate was 15.9% (95% CI 12.6% to 19.6%) (online supplemental table 4) for unpaired single cornea donor transplants, compared with 10.6% for paired transplants (95% CI 7.7% to 14.0%) (online supplemental table 4). Although there was a difference between the two groups (15.9% vs 10.6%) there was an overlap between these confidence intervals suggesting no difference in graft rejection rates (p=0.28) While there was generally no evidence of a difference in incidence between these groups, the results suggested that unpaired single cornea donors had slightly worse rates of rejection and failure (online supplemental table 4).

10.1136/bmjophth-2021-000965.supp4 Supplementary data

There was no evidence of a donor effect associated with 5-year endothelial graft survival when adjusting for transplant and post-transplant risks for PBK or FED (p=0.59 and p=0.17, (online supplemental table 5). There was, however, some evidence of a donor effect for all grafts in the sensitivity analysis for FED, which could suggest a slight difference in outcomes between paired and unpaired donors (p=0.06, online supplemental table 5). In this analysis, however, a test for a donor effect has been performed for eight different models and this increases the chance of a false positive, so this result should be interpreted with caution.

10.1136/bmjophth-2021-000965.supp5 Supplementary data

There was also no evidence of a donor effect associated with 5-year endothelial graft rejection when adjusting for transplant and post-transplant risks for PBK or FED (p>0.99 and p=0.97, respectively) (online supplemental table 5). Similarly, no donor effect was associated with endothelial graft rejection in the sensitivity analysis for FED or PBK.

### Other donor effects for paired cohorts

Overall, none of the donor factors that were included in the PBK and FED models, donor age, ECD and donor–recipient sex match, were associated with endothelial rejection nor endothelial survival up to 5 years post-transplant.

## Discussion

The quality of the donor tissue is an important factor in corneal graft survival. As such, standards are set so that the donor tissue issued by eye banks meets certain quality assurance standards such as above a minimum ECD, endothelial cell quality, and corneal clarity. In the UK, the donor pool is very homogeneous, with a predominance of older white male donors with 51% of donors 70 years or more (mean: 67, median: 70, SD: 13) and 63% male (30% both male and 70 years or more).^4^ This indicates a skew of eye donors to older males in the UK, particularly as the percentage of females in the UK population above 70 years is 7.4% compared with 6.2% for males. Although speculative, this homogeneity may account for the absence of significant donor effects in age and ECD within the ranges used for these variables. We were also not able to investigate donor factors such as the presence of diabetes and other known donor characteristics, as this information is not collected in the registry. While these are limitations when trying to establish an association with donor factors, we were able to investigate a very large dataset. Within these limits, we did not find an influence of recorded donor factors on endothelial graft failure or rejection at 5 years. As there may be other unknown donor effects to consider in paired transplants for the same indication (FED or PBK), a sensitivity analysis, therefore, was performed to establish if this unknown variation was significant across donors. The slight difference in endothelial graft survival for FED patients may indicate that there is some unexplained variation between donors for paired and unpaired groups. It could also suggest that there are donor factors which may not have been identified as contributing to endothelial survival.

ECD is the most critical donor factor in determining graft survival,^13 37^ although there is little evidence of an effect of ECD on graft survival for an ECD of over 2300 cells/mm^2^.^7 8 26–28^ This would support the lack of significance found for ECD in this study. The lower ECDs for unpaired single cornea donors may demonstrate why the alternative cornea was discarded. While unpaired single cornea donors had slightly worse rates of endothelial rejection and failure there was no significant difference in these outcomes between unpaired and paired groups suggesting that single corneal donors and paired corneal donors were comparable, but this may need further research. Although there was a lack of significance, there was a slight difference in endothelial graft rejection in PBK grafts between single cornea donors and paired donor transplants which may need further research.

There is conflicting evidence among studies regarding the influence of donor factors on graft outcome, particularly where the boundaries for donor factors should be, such as donor age, postmortem times. The current available evidence, therefore, is presented in online supplemental table 1. Donor age has been extensively investigated as a potential factor in graft survival and has been reported to have a variable association with graft failure and graft rejection. Williams *et al* reported evidence of an adverse effect of donor age of 80 years or older on graft survival following PK in over 24 000 grafts.^20^ Similarly, arraquer *et al* in a cohort of 895 eyes with 15 years of follow-up found that grafts from donors aged 80 years and older were at a higher risk of failure.^11^ There is evidence that graft survival following DSAEK and DMEK is better for donors under the age of 40 and 50 years.^20^ Much of this variation, however, relates to the donor age boundaries that have been used to compare outcomes and the interplay of other donor and recipient factors. Overall, the evidence would suggest that graft survival is similar for corneas from donors below the age of 65–70 years.^32 65^

Donor and recipient mismatch for either gender or H-Y have been found to have an effect on graft outcome according to the type of transplant and indication. Hopkinson *et al* reported a 40% reduced hazard in FED patients graft survival for female donors to female patients matched transplants compared with H-Y mismatched group (male>female).^73^ The evidence of an H-Y or gender effect for EK, however, is less clear.^7 10 28 31 32^ In our study, however, there was no association between gender mismatch and endothelial rejection or endothelial survival in paired transplants and this was supported by the study of Romano *et al*.^40^ In terms of gender matching, the 2018 report from the Australian Corneal Graft Registry of almost 25 thousand PK grafts showed that grafts from female donors to female recipients had better survival rates than male donors to male recipients or female donors to male recipients.

Our study has several limitations, it largely consisted of donors, aged 61 year and over with the oldest donor 104 years. The intention, however, was to treat elderly FED and PBK patients meaning the median age match (recipient age–donor age) was 1 year but with an IQR of 7–8 years. Donor information collected was limited to age, sex and ECD and we do not have information on other donor characteristics and co-morbidity such as diabetes. Furthermore, some characteristics were not comparable in the paired and unpaired cohorts although to a small extent and in some parts of the analysis. The availability, however, of the UK Transplant Registry offers a unique opportunity to explore donor factors in the UK, which smaller studies or those relying on voluntary data returns may not be able to address. The accuracy, however, of a given analysis can depend on data completeness. In our complete-case study, only 5% were excluded due to incomplete follow-up data.

Overall, we did not identify donor factors that were associated with endothelial rejection and endothelial survival; paired and unpaired transplants had very similar outcomes. There was, however, a borderline significant donor effect in FED patients for endothelial graft survival and would suggest that there may be donor factors which have not been identified and requires further investigation. With regard to the correlation between paired donor transplants, the number of cases with the same cause of failure or rejection was very small so there was no similarity in each transplant outcome. The general lack of difference in endothelial outcomes between paired and unpaired transplants for FED and PBK may reflect a homogeneous donor pool in the UK.

## Data Availability

Data are available on reasonable request. Data can be made available on application to CH, NHS Blood and Transplant.
