# Supplementary material for: Endothelial failure and rejection in recipients of corneas from the same donor
Source: BMJ Open Ophthalmol. 2022 Aug 17;7(1):e000965. doi: 10.1136/bmjophth-2021-000965 (PMC9389126; doi:10.1136/bmjophth-2021-000965)
Supplement: Supplementary data [file bmjophth-2021-000965supp002.pdf]

BMJ Publishing Group Limited (BMJ) disclaims all liability and responsibility arising from any reliance placed on this supplemental material which has been supplied by the author(s)

Table 2. Cause of failure compared for each recipient of paired donors (Group A)

**Cause of failure for recipient 1**	**Cause of failure for recipient 2**									
	Not failed	Final follow-up not reported - failure indicated by regraft	Primary graft failure	Irreversible rejection	Acquired infection	Recurrence of original disease	Endothelial decompensation	Dislocation of EK	Other	Unknown
Not failed	516	24	25	15	4	1	28	1	8	4
Final follow-up not reported - failure indicated by regraft	23	3	0	1	0	0	0	0	0	1
Primary graft failure	19	1	1	1	0	0	2	0		1
Irreversible rejection	14	0	2	1	0	0	2	0	0	0
Acquired infection	4	1	0	0	0	0	1	0	0	0
Recurrence of original disease	0	0	0	0	0	0	0	0	0	0
Endothelial decompensation	31	2	2	1	0	0	7	1	0	0
Dislocation of EK	1	0	0	0	0	0	0	0	0	0
Other	9	0	1	0	0	0	0	0	0	0
Unknown	7	0	0	0	1	0	1	0	0	0
