# Supplementary material for: Endothelial failure and rejection in recipients of corneas from the same donor
Source: BMJ Open Ophthalmol. 2022 Aug 17;7(1):e000965. doi: 10.1136/bmjophth-2021-000965 (PMC9389126; doi:10.1136/bmjophth-2021-000965)
Supplement: Supplementary data [file bmjophth-2021-000965supp003.pdf]

Table 3. Cause of rejection compared for each recipient of paired donors (Group A)					
**Cause of rejection for recipient 1**	**Cause of rejection for recipient 2**				
	No Rejection	Epithelial	Stromal	Endothelial	Not Specified
No Rejection	629	7	4	55	8
Epithelial	8	0	0	0	0
Stromal	4	0	0	1	0
Endothelial	39	1	0	4	0
Not Specified	6	0	0	2	0
