# Supplementary material for: Endothelial failure and rejection in recipients of corneas from the same donor
Source: BMJ Open Ophthalmol. 2022 Aug 17;7(1):e000965. doi: 10.1136/bmjophth-2021-000965 (PMC9389126; doi:10.1136/bmjophth-2021-000965)
Supplement: Supplementary data [file bmjophth-2021-000965supp004.pdf]

Table 4. Five-year endothelial failure and endothelial rejection for FED and PBK transplants (cumulative incidence rates)

	Number of transplants	Number rejected	5-year endothelial rejection rate		Gray's Test P value	Number failed	5-year endothelial failure rate		Gray's Test P value
		N	%	95% CI		N	%	95% CI	
**FED**					**0.99**				**0.37**
**A. Paired**	976	57	8.6	6.5 – 11.1		60	8.3	6.3 – 10.7	
**B. Unpaired (Single cornea donor)**	1011	58	9.9	7.3 – 12.9		77	10.5	8.2 – 13.1	
**C. Unpaired (Double cornea donor)**	4152	246	9.0	7.9 – 10.2		308	9.7	8.6 – 10.8	
**PBK**					**0.28**				**0.88**
**A. Paired**	560	46	10.6	7.7 – 14.0		82	22.3	17.6 - 27.4	
**B. Unpaired (Single cornea donor)**	826	84	15.9	12.6 - 19.6		115	21.7	17.7 - 25.9	
**C. Unpaired (Double cornea donor)**	3313	303	13.7	12.1 - 15.4		450	21.8	19.8 - 23.9	
