# Supplementary material for: Endothelial failure and rejection in recipients of corneas from the same donor
Source: BMJ Open Ophthalmol. 2022 Aug 17;7(1):e000965. doi: 10.1136/bmjophth-2021-000965 (PMC9389126; doi:10.1136/bmjophth-2021-000965)
Supplement: Supplementary data [file bmjophth-2021-000965supp005.pdf]

Table 5. P-values for a donor effect in each risk-adjusted model (adult patients who received a first ocular transplant, using NHSBT supplied corneas from adult paired donors, 1 April 1999 to 31 March 2016 in the UK)

	**FED**	**PBK**
**5-year endothelial graft survival**	0.17	0.59
**Sensitivity analysis 5-year endothelial graft survival**	0.06	0.24
**5-year endothelial graft rejection**	0.97	>0.99
**Sensitivity analysis 5-year endothelial graft rejection**	0.96	>0.99

*Endothelial cell density, donor age group and donor recipient sex match were included in all the models. Each model was also risk-adjusted for significant recipient, transplant, and post-operative factors
