# Supplementary material for: Endothelial failure and rejection in recipients of corneas from the same donor
Source: BMJ Open Ophthalmol. 2022 Aug 17;7(1):e000965. doi: 10.1136/bmjophth-2021-000965 (PMC9389126; doi:10.1136/bmjophth-2021-000965)
Supplement: Supplementary data [file bmjophth-2021-000965supp001.pdf]

Donor Characteristic	Association with	Number of eyes	Study design	Type of graft	Indications	Results endpoint	Reported Outcomes	Results	Author	Year	Country
**Demographics**											
**Age**	Yes	741	Prospective cohort study	PK	Various	20 years	Survival	Per unit increase in donor age showed HR 1.01 (95% CI: 1.0 to 1.03), p= 0.04<br>Higher donor age associated with lower success after the first 5 years (p<0.001)	Anshu et al (16)	2021	Singapore
	Yes	140	Retrospective cohort study	PK	Various	10 years	Survival and failure	Rejection-free survival: <65 years n=107, median= 38.0 months (95% CI:24.1-51.8)<br>≥65 years n= 33, median= 13.0 months (95% CI:5.4-20.5)<br>Overall: p= 0.02 / Overall survival: <65 years n=30, median= 104 months (95% CI: 27.1-80.8)<br>≥65 years n= 18, median= 29.0 months (95% CI:17.4-40.5)<br>Overall: p<0.001<br>Failure: HR= 2.83 (95% CI, 1.261–5.132), p= 0.001	Kwon et al (8)	2020	Korea
	Yes	895	Retrospective cohort study	PK	Various	15 years	Survival	21-40 years versus 61-80 years: p= 0.21<br>41-60 versus 61-80 years: p= 0.95<br>>80 years versus 61-80 years: p= 0.04<br>Further information NA	Barraquer et al (11)	2019	Spain
	PK: Yes<br>DSAEK: Yes<br>DMEK: Yes	PK: 24524<br>DSAEK: 4736<br>DMEK: 1250	Retrospective national database	PK and EK	Various	PK: 30 years<br>DSAEK: 10 years<br>DMEK: 6 years	Survival	PK <50 years n= 6014, HR= 0.79 (95% CI: 0.73-0.86), p<0.001<br>50-59 years n= 4286, HR= 0.92 (95% CI: 0.85-1.01), p= 0.07<br>60-69 years n= 6167, HR= 1.00 p<0.001<br>70-79 years n= 5979, HR= 1.01 (95% CI: 0.94-1.09), p= 0.74<br>≥80 years n= 2078, HR= 1.12 (95% CI: 1.01-1.23), p= 0.03<br>Overall: p<0.001<br>DSAEK: Better survival from donors <40 years age versus 40-69 years (p= 0.01) and ≥70 years (p= 0.001), p= 0.01<br>DMEK: Significantly poorer survival from donors <50 years versus 60-69 years (p= 0.001) and 70-79 years (p= 0.004), p= 0.01<br>Further information NA	Williams et al (20)	2018	Australia
	No	1330	Cohort study within a multicenter, double-masked, randomized clinical trial	DSAEK	FED, ABK and PBK	3 years	Survival and failure	Survival: ≤30 years: n= 67 (1.7%), 31-50 years n= 214 (5.4%), 51-60 years n=357 (6.7%)<br>61-70 years n=489 (6.5%), 71-75 years n= 203 (7.0%)<br>HR= 1.17 per decade (99% CI: 0.89-1.53), p= 0.15<br>Primary Failure: Age range ≤30- 71-75 years, OR= 1.27 (99% CI: 0.85-1.88) per decade, p= 0.12	Terry et al (21)	2018	USA
	No	1639	Retrospective cohort study	DSAEK	FED, Bullous keratopathy and [illegible]	2 years	Survival	HR= 1.0 (95% CI: 0.99–1.01), p= 0.32, Further information NA	Potapenko et al (22)	2017	Sweden
	No	9415	Retrospective case series	PK	FED, PBK and other excluding KC	5 years	Survival	Three age groups: 0-60 years, 61–75 years and 76– 90 years, p= 0.91<br>Further information NA	Wakefield et al (23)	2016	UK
	PK: Yes<br>DSAEK: No	PK: 8281<br>DSAEK: 1801	Retrospective national database	PK and DSAEK	Various	PK: 9 years<br>DSAEK: 6 years	Survival	PK <60 years mean= 9.53 years (95% CI: 9.21-9.85), median 11.42 years<br>>60 years mean= 7.83 years (95% CI: 7.54-8.12), median 8.27 years<br>Overall p<0.001<br>DSAEK p= 0.12	Williams et al (24)	2015	Australia
	Yes	377	Retrospective cohort study	PK	Various	1 year	Failure	Donors stratified by 10 year age groups, range 0-99 years<br>Failure mean= 49.6 ± 16.6 years<br>Non-failure mean= 42.8 ± 16.2 years<br>Total mean= 44.6 ± 16.4 years<br>HR= 1.26 (95% CI: 1.14-1.44), p<0.001<br>Donors startified by 5 year age groups	Yu et al (13)	2014	Germany
	No	176	Multi-center, prospective, double-masked clinical trial	PK	Various	10 years	Survival	12-65 years= 77% n= 125, 66-75 years= 71% n= 51, p= 0.11<br>Older donor age (as a continuous variable) was associated with slightly lower graft success after the first 5 years (p<0.001)	Lass et al (25)	2013	USA
	No	1090	Multicenter, prospective, double-masked clinical trial	PK	Various	10 years	Survival	12-65 years (n= 707): 77%, 12-33 years (n= 80): 96%<br>66-75 years (n= 383): 71%, 72-75 years (n= 130): 62%<br>Evidence of a donor age effect at the extremes of the age range, difference= +6% (95% CI: -1 to +12), p= 0.11<br>As a continuous variable, higher donor age was associated with lower success beyond the first 5 years (p<0.001)	Mannis et al (26)	2013	USA
	No	1629	Retrospective national database	PK	Various	1 year	Survival	10-year intervals: highest 93.1% donor age 21–30 years, lowest 85.7% donor age 81–85 years<br>Survival 81–85 years= 85.7% versus <81 years= 88.0%, OR= 1.01 (95% CI 0.99-1.02), p= 0.2	Patel et al (9)	2011	New Zealand
	Yes	885	Retrospective case series	PK	Various	5 years	Survival	<45 years= 91.9% n=171, 45-54 years= 82.2% n=251<br>55-59 years= 89.4% n=174, ≥60 years= 69.1% n=314<br>HR 1.24 (95% CI:1.13, 1.36), per +5 years: p= 0.01	Wagoner et al (15)	2010	Saudi Arabia
	No	1090	Multicenter prospective, double-masked, controlled clinical trial	PK	FED and PBK	5 years	Survival	12-65 years= 86%, 66-75 years= 86%,<br>Difference= 0%, upper limit of one-sided 95% CI: 4%, p=0.11	Robin et al (65)	2008	USA
	No	229	Multicenter, prospective, double-masked clinical trial	PK	FED and PBK	5 years	Survival	Weak negative association between ECD and donor age analysed as a continuous variable (r [adjusted for baseline ECD]= −0.19 (95% CI: −0.29- −0.08)<br>12-65 years n=162 baseline ECD= 2679 cells/mm² (IQR 2446-2905), 5-year median cell loss= 824 cells/mm² (69% IQR 613-1342)<br>66-75 years n= 67 baseline ECD= 2562 cells/mm² (IQR 2414-2798), 5-year median cell loss= 654 cells/mm² (75%, IQR 538-986)	Lass et al (27)	2008	USA
	Overall: Yes<br>KC: No	13341	Retrospective national database	PK	Various	18 years	Survival	Total cohort in 20 year groups p<0.00001<br>KC only mean survival: 0-20 years 15.07 years (95% CI: 14.28-15.86)<br>21-40 years 16.20 years 69.6% (95% CI: 15.07-17.34)<br>41-60 years 15.81 years (95% CI: 15.24-16.37)<br>61-80 years 16.33 years (95% CI: 15.78-16.89)<br>>80 years 15.88 years (95% CI: 15.01-16.75) / All excluding KC mean survival: 0-20 years 10.13 years (95% CI: 9.31-10.94)<br>21-40 years 11.23 years (95% CI: 10.22-2.24)<br>41-60 years 10.71 years (95% CI: 9.97-11.45)<br>61-80 years 10.02 years (95% CI: 9.51-10.53)<br>>80 years 8.93 years (95% CI: 7.93-9.93)	Williams et al (28)	2007	Australia
	No	116	Retrospective cohort study	PK	Various	5 years	Failure	Continuous age category: adjusted HR= 1.00 (95% CI: 0.98-1.02), p= 0.68,<br><65 versus ≥65 years: adjusted HR= 1.17, p= 0.68<br><60 versus >70 years: adjusted HR= 2.1, p= 0.09<br>All HRs>1.00 for older versus younger groups	Al-Muammar et al (29)	2005	Canada
	No	179	Prospective interventional study	PK	Various	2 years	Survival and rejection	Survival: <85 years: 87.4% n= 143, >85 years: 80.6% n= 36, p= 0.2<br>Rejection: <85 years: 16% n= 23, >85 years: 33% n= 12, p= 0.02	Gain et al (30)	2002	France
	No	7741	Retrospective cohort study	PK and lamellar keratoplasty	Various	8 years	Survival	<10 years: 81% n= 59, 11-20 years: 74% n= 275<br>21-30 years 76% n=67% n= 314, 31-40 years 82% n= 287<br>41-50 years: 72% n= 537, 51-60 years: 66% n= 930<br>61-70 years: 68% n= 1567, 71-80 years: 61% n=1476<br>81-90 years: 64% n= 318, >91 years: 72% n=17<br>p= 0.10	Williams et al (31)	1997	Australia
	No	58	Prospective matched cohort	PK	Various	5 years	Rejection and failure	Paediatric donors (0-5 years) and adult donors (40-70 years)<br>Rejection: Paediatric n= 20/29 (69%), Adult n= 5/29 (17.2%)<br>Failure: p= 1.0	Palay et al (32)	1997	USA
	No	93	Retrospective case series	PK	Various	2 years	Survival and failure	Clear grafts: mean age= 45.2 years (SD= 20.34)<br>Unclear grafts: mean age= 47.9 years (SD= 18.81)<br>Mean age of all grafts= 45.8 years<br>Further information NA	Chang et al (33)	1996	USA
	Yes	698	Retrospective case series	PK	Various	5 years	Survival	Range 3-94 years, Mean±SD= 63.5±16.3 years<br>HR 1.3 in proportion to each 10-years in age, p<0.01	Yamagami et al (34)	1996	Japan
	No	539	Retrospective observational study	PK	Various	30 months	Failure	Median age= 45 years<br>Failue with older donor age: RR= 1.0 (95% CI:1.0-1.0), p= 0.80 as a continuous variable.<br>No significant association was found when analyzed as a categorical variable. Further information NA	Boisjoly et al (35)	1993	Canada
	No	63	Prospective case control	PK	Not specified	5 years	Survival	≤65 years: 72%, n=26, mean 55.8 years, range 23-65 years<br>>65 years: 86.3%, n=37, mean 75.7 years, range 66-104 years, Not significant<br>Further information NA	Andersen and Ehlers (36)	1988	Denmark
	Yes	231	Prospective interventional study	PK	Various	1 year	Survival and failure	Mean age= 70.8 (SD 12.5), range= 42-95 years<br>Survival: <80 years= 81.7%, >80 years= 91.3%<br>Failure RR= 4.3 (95% CI: 1.5-11.8) for donors <80 years versus >80 years, p= 0.02	Borderie et al (37)	1988	France
	Yes	1069	Retrospective cohort study	PK	Various	5 years	Survival	≤15 years n= 47, 16-65 years n= 608, ≥65 years n= 414, p= 0.002<br>Further information NA	Völker-Dieben et al (38)	1987	The Netherlands
**Sex**	No	140	Retrospective cohort study	PK	Various	10 years	Survival	Rejection-free survival: Female n= 59, median= 38.0 months (95% CI: 20.9-55.0)<br>Male n= 81, median= 24.0 months (95% CI: 11.9-36.0)<br>p= 0.35 / Overall survival: Female n= 20, median= 104 months<br>Male n= 28, median= 85 months (95% CI: 31.7-138.2)<br>p= 0.77	Kwon et al (8)	2020	Korea
	No	893	Retrospective cohort study	PK	Various	15 years	Failure	HR>1, p= 0.05, Further information NA	Barraquer et al (11)	2019	Spain
	Yes	1330	Cohort study within a multicenter, double-masked, randomized clinical trial	DSAEK	FED, ABK and PBK	3 years	Rejection	Female n= 486, HR= 2.02 (95% CI: 0.92-4.45)<br>Male n= 844, HR= 1<br>p= 0.02	Stulting et al (66)	2018	USA
	No	1330	Cohort study within a multicenter, double-masked, randomized clinical trial	DSAEK	FED, ABK and PBK	3 years	Failure	Female n= 486, Male n= 844, female OR= 1.54 (99%CI: 0.67-3.51), p= 0.18	Terry et al (21)	2018	USA
	PK: Yes<br>DMEK: No	PK: 24201<br>DMEK: 1250	Retrospective national database	PK and DMEK	Various	PK: 30 years<br>DMEK: 6 years	Survival	PK: Male n=15317, Female n= 8884, p= 0.01<br>DMEK: Male n=786, Female n= 464, p= 0.06<br>Further information NA	Williams et al (20)	2018	Australia
	No	PK: 8279<br>DSAEK: 1801	Retrospective national database	PK and DSAEK	Various	PK: 9 years<br>DSAEK: 6 years	Survival	PK mean survival: Female: 8.82 years (95% CI: 8.44-9.20)<br>Male: 8.49 years (95% CI: 8.22-8.75)<br>p= 0.45 / DSAEK mean survival: Female= 4.06 years (95% CI: 3.71-4.42)<br>Male= 3.95 years (95% CI: 3.73-4.17)<br>p= 0.53	Williams et al (24)	2015	Australia
	Yes	3014	Retrospective cohort study	PK	Various	5 years	Failure	Female n= 1212 HR= 1.0, Male n= 1802 HR= 1.3 (95% CI: 1.1-1.5), p= 0.01	Armitage et al (39)	2014	UK
**Donor/recipient sex match/mismatch**	No	4341	Retrospective cohort study	DSAEK	FED	2 years	Rejection and failure	Rejection: n=175 (4%)<br>Male to female HR= 1<br>Male to male: HR= 1.32 (95% CI: 0.89-1.96)<br>Female to male: HR= 1.76 (95% CI: 1.15-2.70)<br>Female to female: HR= 1.26 (95% CI: 0.83-1.93)<br>Overall p=0.12<br>Rejection leading to failure: n=58 (1.3%) / Failure: n=477 (11%)<br>Male to female HR= 1<br>Male to male: HR= 1.03 (95% CI: 0.83-1.30)<br>Female to male: HR= 1.15 (95% CI: 0.89-1.59)<br>Female to female: HR=1.19 (95% CI: 0.94-1.49)<br>Overall p=0.28	Romano et al (40)	2021	UK / Italy
	Yes	24827	Retrospective national database	PK	Various	30 years	Survival	Female to female n= 4269, HR= 0.85 (95% CI: 0.78 - 0.93), p<0.001<br>Female to male n= 4615, HR= 0.96 (95% CI: 0.88-1.04), p= 0.28<br>Male to female n= 7222, HR= 0.93 (95% CI: 0.87-1.00), p= 0.06<br>Male to male n= 8095, HR= 1.00<br>Not available n= 626 HR= 0.90 (95% CI: 0.74-1.10), p= 0.3<br>Female to female better survival than male to male (p<0.001) or female to male (p= 0.02)	Williams et al (20)	2018	Australia
	Yes	1789	Retrospective cohort study	DSAEK	FED, Bullous keratopathy and retransplantation	2 years	Failure	Female n=37 of 688, HR= 1<br>Male n= 79 of 1023, HR 1.5 (95% CI: 1.04–2.28), p= 0.03<br>Female to male: n= 26 of 410, HR= 1<br>Female to male: n= 11 of 278, HR= 0.5 (95% CI: 0.23-1.15), p= 0.11<br>Male to female: n= 48 of 636, HR= 1.3 (95% CI: 0.77-2.23), p= 0.32<br>Male to male: n= 31of 387, HR= 1.2 (95% CI: 0.82-2.20) p= 0.46	Potapenko et al (22)	2017	Sweden
	No	DMEK: 705<br>DSAEK : 1312	Retrospective cohort study	DMEK and DSAEK	FED	5 years	Survival and rejection	Female versus male donors<br>Rejection episodes: p= 0.07 for female recipients, p= 0.33 for male recipients<br>Survival: p= 0.67 for female recipients, p= 0.17 for male recipients	Price et al (67)	2018	USA
	Yes	4046	Retrospective cohort study	PK, DALK and EK	Various	5 years	Survival	Male to female 74% (CI:71-77%)<br>Female to female 82% (CI: 79– 85%)<br>Female to male 78% (CI: 73– 82%)<br>Male to male 78% (CI: 74– 81%)<br>Overall p= 0.0001<br>Study duration: 13 years (1999-2012)	Hopkinson et al (74)	2017	UK
**H-Y, HLA-A1 and HA-3 match**	No	4341	Retrospective cohort study	DSAEK	FED	2 years	Rejection and failure	Rejection: n=175 (4%)<br>H-Y matched HR= 1<br>HY mismatched HR= 0.71 (95%CI: 0.51-0.98)<br>Overall p=0.06<br>Rejection leading to failure: n=58 (1.3%) / Failure: n=477 (11%)<br>H-Y matched HR= 1<br>HY mismatched: HR= 0.89 (95%CI: 0.75-1.07)<br>Overall p=0.14	Romano et al (40)	2021	UK / Italy
	H-Y: Yes<br>HLA-A1: No<br>HA-3: No	229	Prospective interventional study	PK	Various	2 years	Survival	Rejection-free survival<br>H-Y matched versus. mismatched= 88% versus 77%, RR= 2.07 (95% CI: 1.10–3.88), p= 0.02<br>HA-3 matched versus mismatched= 85% versus. 73%, RR= 1.65 (95% CI: 0.36–7.62), p= 0.52<br>HLA-A1 match versus. mismatch RR= 0.86 (95% CI: 0.46–1.63), p= 0.86	Böhringer et al (41)	2006	Germany
	H-Y: no	396	Retrospective cohort study	PK	Various	4 years	Failure and rejection	Male to male n= 135, Female to male n=107, Female to female n= 60, Male to female n=94<br>Failure: RR= 0.75, p= 0.23<br>Rejection: RR= 0.99, p= 0.97	Inoue et al (42)	2000	Japan
**HLA-A and HLA-B**	Yes	303	Retrospective cohort study	PK	Various	4 years	Survival	No mismatches= longer survival, HLA-A : p= 0.01, HLA-B : p= 0.03<br>Fewer splint antigen mismatches OR= 0.41 (95% CI: 0.23 – 0.73)	Bartels et al (43)	2003	The Netherlands
	Yes	459	Retrospective case series	PK	Various	10 years	Rejection	Donor-recipient match of ≥2 alleles in HLA-A, -B or -DR reduces rejection rate by at least 10% in low-risk (10 years after PK; p<0.04) and 40% in high-risk patients (3 years after PK; p<0.0001)<br>HLA-B mismatches are important prognostic factors for both low- (p<0.008) and high-risk patients (p<0.003)	Khaireddin et al (44)	2003	Germany
	Yes	115	Prospective case series	PK	NA	5 years	Survival and failure	Frequencies of the HLA-A26, -B35 and -B44 alleles in donors were increased in recipients who developed graft failure<br>Survival similar when one or two A26, B35 or B44 alleles were present on the donor<br>When both a B-locus match and an A26, B35 or B44 allele were present, the negative effect on survival was twice as strong, p<0.001	Creemers et al (45)	1999	South Africa
	Class 1: No<br>Class 2: [illegible]	542	Prospective case series	PK	NA	15 months	Survival	Rejection-free survival: Class 1: no mismatch n= 44, RR= 1.00<br>Per mismatch RR= 1.27 (95% CI: 0.97-1.67) / Rejection-free survival: Class 2: no mismatch n= 174, RR= 1.00<br>Mismatch identified n= 368, RR= 0.58 (95% CI: 0.37-0.92)	Vail et al (46)	1994	UK
	No	37	Prospective case control	PK	NA	3 years	Survival and rejection	No effect on overall graft survival, the incidence of irreversible rejection, or the incidence of rejection episodes<br>Further information NA	Fink et al (47)	1994	USA
	Yes	539	Retrospective observational study	PK	Various	30 months	Failure	Two mismatched antigens: RR=1.5 (95%CI: 1.0-2.3), p=0.08<br>Further information NA	Boisjoly et al (35)	1993	Canada
	Yes	740	Retrospective cohort study	PK	Various	5 years	Survival	Matched n=497 versus not matched n=721, p= 0.0008<br>Further information NA	Völker-Dieben et al (38)	1987	The Netherlands
	Yes	97	Prospective, single-center, masked study	PK	NA	2 years	Survival	Matched: 80.1%, Unmatched: 38.5%, RR= 11.2, p<0.02<br>Further information NA	Sanfilippo et al (48)	1986	USA
	Yes	250	Retrospective case series	PK	Herpes Simplex Keratitis	3 years	Survival	Vascularised recipients: Matched 67.2% n=110, Unmatched 50.3% n=140, p=0.04<br>Further information NA	Völker-Dieben et al (49)	1984	The Netherlands
	Yes	324	Prospective case series	PK	Various	1 year	Survival	Vascularised recipients (non-immunological failures excluded): Matched 88.3%, n=113, Unmatched 78.5%, n=157, p=0.03<br>Number of HLA mismatches: 0= 91% n=19, 1= 87% n=53, 2= 85.4% n=41	Völker-Dieben et al (50)	1982	The Netherlands
**Cause of death**	PK: Yes<br>DSAEK: No	PK: 23712	Retrospective national database	PK and DSAEK	Various	PK: 30 years	Survival	Trauma had significantly better survival than any of the other four major causes of death (all p<0.001)<br>p<0.001 (not an independent factor in multivariate analysis)	Williams et al (20)	2018	Australia
	PK: Yes<br>DSAEK: No	PK: 8244<br>DSAEK: 1783	Retrospective national database	PK and DSAEK	Various	PK: 9 years<br>DSAEK: 6 years	Survival	PK mean survival: Cardiac event: 8.60 years (95% CI: 8.22, 8.98)<br>Malignancy: 7.82 years (95% CI: 7.43, 8.21)<br>Trauma: 9.59 years (95% CI: 9.04, 10.14)<br>Respiratory event: 7.81 years (95% CI: 7.20, 8.43)<br>Intracranial/cerebral haemorrhage: 8.38 years (95% CI: 8.01, 8.76)<br>Other causes: 9.28 years (95% CI: 8.56, 10.00)<br>Overall p<0.001 (not an independent factor in multivariate analysis)	Williams et al (24)	2015	Australia
	No	1629	Retrospective national database	PK	Various	1 year	Survival	Range: 84.0%–90.6%, p= 0.27<br>Further information NA	Patel et al (9)	2011	New Zealand
	No	18181	Retrospective national database	PK	Various	15 years	Survival	Mean survival: Cerebrovascular: 11.98 years (95% CI: 11.38-12.57)<br>Malignancy: 11.75 years (95% CI: 11.12-12.38)<br>Trauma: 12.63 years (95% CI: 12.06-13.19)<br>Respiratory: 12.55 years (95% CI: 11.91, 13.19)<br>Cardiac/circulatory: 12.11 years 72% (95% CI: 11.60, 12.62)<br>Other: 12.89 years (95% CI: 11.97, 13.81)<br>Overall p<0.00001<br>Excluding donors from trauma p=0.4	Williams et al (28)	2007	Australia
**Multi-organ donor**	No	24827	Retrospective national database	PK	Various	30 years	Survival	Eye-only donor n=22684, Multi-organ n=2143, p<0.001<br>(variable not retained in the multivariate analysis suggesting it is not an independent factor)<br>Further information NA	Williams et al (20)	2018	Australia
	PK: Yes<br>DSAEK: No	PK: 8301<br>DSAEK: 1801	Retrospective national database	PK and DSAEK	Various	PK: 13 years<br>DSAEK: 6 years	Survival	PK mean survival: No: 8.48 years (95% CI: 8.25, 8.70)<br>Yes: 9.00 years (95% CI: 8.56, 9.45)<br>p<0.001 (not an independent factor in multivariate analysis) / DSAEK mean survival: No: 4.10 years (95% CI: 3.89, 4.32)<br>Yes: 3.37 years (95% CI: 2.95, 3.79), p= 0.50<br>Further information NA	Williams et al (24)	2015	Australia
	Yes	13350	Retrospective national database	PK	Various	15 years	Survival	Mean survival: Cadaveric donors: 12.36 years (95% CI: 12.05-12.66) n=12590<br>Multi-organ donors: 11.23 years (95% CI: 10.35-12.10) n=760<br>p=0.0003<br>Further information NA	Williams et al (28)	2007	Australia
**Race**	No	140	Retrospective cohort study	PK	Various	10 years	Survival	Rejection-free survival: Asian n= 20, median= 19 months (95% CI: 9.8-28.1)<br>Others n= 98, median= 30.0 months (95% CI: 17.5-42.4)<br>p= 0.65 / Overall survival: Asian n= 10, median= 38 months (95% CI: 0-83.2)<br>Others n= 31, median= 104.0 months<br>p= 0.33	Kwon et al (8)	2020	Korea
**ABO blood group compatibility**	Yes	37	Prospective case control	PK	NA	3 years	Failure	Failure due to rejection: ABO-incompatible group 30%, ABO-compatible group 16%, RR= 1.98 (95% CI: 1.15-3.13)	Fink et al (47)	1994	USA
	No	1002	Multi-center prospective, double-masked, clinical trial	PK	FED and PBK	5 years	Rejection and failure	Failure due to rejection: ABO recipient-donor compatibility: n= 32 (6%)<br>ABO incompatibility: n= 12 (4%), HR= 0.65 (95% CI: 0.33-1.25) / Incidence of rejection episode: ABO compatible: n= 64 (12%)<br>ABO incompatible: n= 25 (8%)	Dunn et al (51)	2009	USA
**Crossmatching**	No	759	Prospective case series	PK	NA	2 years	Failure	Positive crossmatching n= 61 (8%) failure n=10 (16%)<br>Negative crosshatching n= 698 (92%) failure n= 210 (30%)	des Marchais et al (52)	1998	Canada
**Lens status**	No	1629	Retrospective national database	PK	Various	1 year	Survival	Phakic= 88.6%, 1374 of 1551, Pseudophakic= 81.8%, 31 of 38, Aphakic= 91.6%, 11 of 12, p= 0.80	Patel et al (9)	2011	New Zealand
**Tissue Characteristics**											
	Yes	741	Prospective cohort study	PK	Various	20 years	Survival	Per unit increase in donor endothelial cell count showed HR 0.9991 (95%CI:0.99996-0.99996) p= 0.03<br>Further information NA	Anshu et al (16)	2021	Singapore
	PK: Yes<br>DSAEK: Yes	PK: 5031<br>DSAEK: 3205	Retrospective national database	PK and DSAEK	Various	PK: 10 years<br>DSAEK: 10 years	Survival	PK: <2500 cells/mm² poorer survival versus 2750-2999 cells/mm², 3000-3249 cells/mm², or ≥3500 cells/mm² (all p<0.001), p<0.001<br>DSAEK: <2500 cells/mm² poorer survival versus 2500-3249 cells/mm² or ≥3250 cells/mm² (both p<0.001)	Williams et al (20)	2018	Australia
	PK: No<br>DSAEK: Yes	PK: 2080<br>DSAEK: 1039	Retrospective national database	PK and DSAEK	Various	PK: 7 years<br>DSAEK: 6 years	Survival	PK mean survival: <2500 cells/mm²= 5.24 years (95% CI: 4.74-5.75)<br>2500 – 2999 cells/mm²= 5.57 years (95% CI: 5.29-5.86)<br>3000 – 3499 cells/mm²= 5.36 years (95% CI: 5.05-5.66)<br>≥3500 cells/mm²= 5.31 years (95% CI: 4.96-5.66)<br>Overall p= 0.28 / DSAEK mean survival: <2500 cells/mm²= 2.00 years (95% CI: 1.59-2.42)<br>2500 – 2999 cells/mm²= 3.72 years (95% CI: 3.31-4.12)<br>3000 – 3499 cells/mm²= 3.62 years (95% CI: 3.33-3.91)<br>≥3500 cells/mm²= 3.28 years (95% CI: 2.66-3.89)<br>Overall p= 0.004	Williams et al (24)	2015	Australia
	Yes	377	Retrospective cohort study	PK	Various	1 year	Failure	Total mean= 2694 cells/mm², Failure mean= 2648 cells/mm², HR 0.80 (95% CI: 0.68–0.94), p=0.008<br>Lower ECD a significant factor for graft failure, HR= 0.99, p= 0.004<br>No significant difference in the influence on graft failure between ≤3000 cells/mm² and >3000 cells/mm²	Yu et al (13)	2014	Germany
	Yes	231	Prospective case series	PK	Various	1 year	Survival	Mean: 2336 cells/mm² (SD 381), Range: 1557-3546 cells/mm²,<br><2000 cells/mm²= 76.1%, >2000 cells/mm²= 86.6%, p= 0.05<br>ECD effect not significant in mulitvariate analysis	Borderie et al (37)	1988	France
	No	140	Retrospective cohort study	PK	Various	10 years	Survival	Rejection-free survival: <2700 cells/mm² n=63 median 38.0 months (95% CI:23.3-52.6)<br>≥2700-<3000 cells/mm² n=44 median 23.0 months (95% CI: 9.1-36.8)<br>≥3000 cells/mm² n=33, median 19.0 months (95% CI: 3.0-34.9)<br>p= 0.73 / Overall survival: <2700 cells/mm² n=19, median 85.0 months (95% CI: 38.0-131.9)<br>≥2700-<3000 cells/mm² n=17, median 41.0 months<br>≥3000 cells/mm² n=12<br>p= 0.48	Kwon et al (8)	2020	Korea

**Baseline endothelial cell density (ECD)**	No	1330	Cohort study within a multicenter, double-masked, randomized clinical trial	DSAEK	FED, ABK and PBK	3 years	Survival	<2500 cells/mm²: n=301<br>2500-<2750 cells/mm²: n=476<br>2750-<3000 cells/mm²: n= 303<br>≥3000 cells/mm²: n= 250<br><br>OR= 0.57 (99% CI: 0.24-1.36) p= 0.15, per 500 cells	Terry et al (21)	2018	USA
	No	1639	Retrospective cohort study	DSAEK	FED, Bullous keratopathy and retransplantation	2 years	Failure	<2300 cells/mm²: n= 17 of 352, HR= 1.3 (95% CI: 0.76-2.35)<br>≥2300 cells/mm² : n=95 of 1287, HR= 1<br>p= 0.31	Potapenko et al (22)	2017	Sweden
	No	9415	Retrospective case series	PK	FED, PBK and other excluding KC	5 years	Survival	≤2400->2600 cells/mm², HR= 0.3-3.3, p= 0.6<br>Further information NA	Wakefield et al (23)	2016	UK
	No	1629	Retrospective national database	PK	Various	1 year	Survival and failure	Survival: mean= 3020 cells/mm², SD= 334 cells/mm²<br>Failure: mean= 3013 cells/mm², SD= 330 cells/mm²<br>p= 0.77<br><br>Divided in 300 cells/mm² intervals: survival range= 87.1% – 90.7%, p= 0.94	Patel et al (9)	2011	New Zealand
	No	500	Retrospective cohort study	PK	FED and PBK	5 years	Survival and failure	Failure n= 17 (3.4%), median= 2670 cells/mm²<br>Nonfailure n= 483 (96.6%), median= 2687 cells/mm², p= 0.91	Lass et al (53)	2010	USA
	No	885	Retrospective case series	PK	Various	5 years	Survival	<2500cells/mm²= 78.7% n=263, 2500-2599 cells/mm²= 84.3% n= 156<br>2600-2899cells/mm²= 79.9% n=270, ≥2900cells/mm²= 82.6% n=221<br>HR= 0.96 (95% CI: 0.91-1.01) (per +100 cells/mm²), p= 0.10	Wagoner et al (15)	2010	Saudi Arabia
	No	1090	Retrospective cohort study	NA	FED and PBK	5 years	Failure	2300-<2500 cells/mm² n=318 (99% CI:14%±5%)<br>2500-<2700 cells/mm² n=270 (99% CI: 17%±6%)<br>2700-<3000 cells/mm² n=361 (99% CI: 13%±5%<br>≥3000 cells/mm² n=141 (99% CI: 9%±7%)	Sugar et al (54)	2009	USA
	No	64	Retrospective case series	PK	Various	2 years	Survival	<2700 n= 28 (85%), ≥2700 n=25 (81%), p= 0.58, Further information NA	Chang et al (33)	1996	USA
**Polymorphism/Polymegathism**	No	1090	Retrospective cohort study	PK	FED and PBK	5 years	Failure	None n= 951 (99% CI: 13%±3%), Mild (slight) / Moderate n= 139 (99% CI: 16%±9%), Further information NA	Sugar et al (54)	2009	USA
**Peripheral endothelial cell**	No	1090	Retrospective cohort study	PK	FED and PBK	5 years	Failure	Diffuse 99% CI: 9%±13%, Peripheral 99% CI: 8%±10%, Further information NA	Sugar et al (54)	2009	USA
**Defect on <50% of epithelium**	No	1090	Retrospective cohort study	PK	FED and PBK	5 years	Failure	99% CI: 15%±4%, Further information NA	Sugar et al (54)	2009	USA
**Moderate epithelial haze**	No	1090	Retrospective cohort study	PK	FED and PBK	5 years	Failure	99% CI: 19%±17%, Further information NA	Sugar et al (54)	2009	USA
**Moderate epithelial**	No	1090	Retrospective cohort study	PK	FED and PBK	5 years	Failure	99% CI: 10%±5%, Further information NA	Sugar et al (54)	2009	USA
**Mild stromal oedema**	No	1090	Retrospective cohort study	PK	FED and PBK	5 years	Failure	99% CI: 14%±6%, Further information NA	Sugar et al (54)	2009	USA
**Arcus**	No	1090	Retrospective cohort study	PK	FED and PBK	5 years	Failure	99% CI: 12%±3%, Further information NA	Sugar et al (54)	2009	USA
**Descemet folds**	No	1090	Retrospective cohort study	PK	FED and PBK	5 years	Failure	99% CI: 14%±4%, Further information NA	Sugar et al (54)	2009	USA
**Snail tracks**	No	1090	Retrospective cohort study	PK	FED and PBK	5 years	Failure	Central: 99% CI: 19%±12%, Peripheral: 99% CI: 8%±5%, Diffuse: 99% CI: 17%±8%, Further information NA	Sugar et al (54)	2009	USA
**Rigidity of cornea**	No	377	Retrospective cohort study	PK	Various	1 year	Failure	Surgeon assessment on a linear scale of 0= rigid to 5= soft, Total mean= 2.5, Failure mean= 2.6, HR= 0.99 (95% CI:0.72-1.35), p= 0.93	Yu et al (13)	2014	Germany
**Clarity of cornea**	No	377	Retrospective cohort study	PK	Various	1 year	Failure	Surgeon assessment on a linear scale of 0= clear to 5= blurred, Total mean= 2.0, Failure mean= 2.0, HR= 0.92 (95% CI:0.61-1.38), p= 0.69	Yu et al (13)	2014	Germany
**Co-morbidities**											
**Prior glaucoma surgery**	No	1330	Cohort study within a multicenter, double-masked, randomized clinical trial	DSAEK	FED, ABK and PBK	3 years	Survival	Yes= 80.1%, n=31<br>No= 94.1%, n=1299<br>OR= 2.82 (99% CI: 0.44-18.15), p= 0.15	Terry et al (21)	2018	USA
**Diabetes**	Yes	1330	Cohort study within a multicenter, double-masked, randomized clinical trial	DSAEK	FED, ABK and PBK	3 years	Failure	Yes: n= 357, No: n= 973<br>Failue: OR= 2.35 (99% CI: 1.03-5.33), p= 0.01	Terry et al (21)	2018	USA
	No	183	Retrospective case series	PK and DSAEK	Various	6 years	Failure	All grafts: PK n= 47 (25.7%), DSAEK n= 136 (74.3%), Diabetic versus non-diabetic HR= 1.69 (95% CI: 0.56-5.06), p= 0.35<br>Diabetic: PK: n= 1 (4.1%), DSAEK: n= 3 (12.5%), Non-diabetic: PK: n= 6 (3.8%), DSAEK: n= 12 (7.5%)	Vislisel et al (55)	2015	USA
	No	1090	Multicenter, prospective, double-masked, controlled clinical trial.	PK	FED, ABK and PBK	10 years	Failure	Diabetic= 23% (95% CI: 16%-30%) n= 199, Non-diabetic= 26% (95% CI: 23%-29%) n= 891<br>10-year failure difference= −2% (95% CI: −10%- +6%), p= 0.60	Lass et al (56)	2015	USA
**Chronic kidney disease**	No	40	Retrospective observational study	PK and EK	Various	6 months	Failure	PK: n= 18 (46.2%), DSAEK: n= 16 (41%), DMEK: n= 5 (12.8%)<br><br>Primary failure: PK n=1 (5.1%) EK n=1 (5.3%)<br>Secondary failure: PK n= 3 (16.7%), EK: n=2 (10.5%)	Sravani et al (57)	2020	India
**Anticorneal antibodies**	No	100	Prospective interventional single centre study	PK	Various	5 years	Rejection	Donor specific anticorneal stroma IgM found in 28% of recipients before transplantation. This pre-immunisation was not associated with a higher risk of rejection<br>Anti-donor stromal lamellae IgM found in 14 recipients. Anti-donor keratocyte IgM present in 28 recipients	Borderie et al (68)	2004	France
**Tissue Preparation**											
**Harvest Technique**	No	454	Retrospective case series	PK	NA	10 years	Failure	Primary failure: Enucleation: n= 1 (0.0006%), In situ corneoscleral disc excision n= 6 (0.004%), p>0.05	Filev et al (10)	2018	Germany
	No	229	Retrospective case series	PK	KC , corneal scars and dystrophies	10 months	Failure	Primary failure: Morgue trephination n= 3/108 (2.7%), sclerocorneal removal technique n= 2/121 (1.6%), p= 0.19	Yuksel et al (12)	2015	Turkey
**Graft diameter**	No	1629	Retrospective national database	PK	Various	1 year	Failure	Small ≤7.25mm OR= 2.41 (95% CI:1.50–3.89), p=0.01<br>Large ≥8.5 mm OR= 1.88 (95% CI 1.18–2.60), p<0.01	Patel et al (9)	2011	New Zealand
	Yes	1218	Retrospective cohort study	PK	Various	5 years	Survival	Small ≤7.1mm n= 430, medium 7.2-7.9mm n= 448, large ≥8.0mm n= 340, p<0.00001<br>Further information NA	Völker-Dieben et al (38)	1987	The Netherlands
	Yes	97	Prospective, single-center, masked study	PK	NA	2 years	Rejection	≥8.0mm had a significantly lower rejection rate p= 0.02<br>6.1-7.0mm had the highest rejection rate<br>Further information NA	Sanfilippo et al (48)	1986	USA
	Yes	520	Retrospective cohort study	PK	Various	1 year	Survival	7.0-8.4mm= 82% n=474, 8.5-8.9mm= 57% n=31, >9.0mm= 56% n=15, p<0.005<br>Further information NA	Völker-Dieben et al (59)	1982	The Netherlands
**Death to enucleation time**	No	895	Retrospective cohort study	PK	Various	15 years	Failure	<2 versus 2-4 hours: p= 0.9<br>4-6 versus 2-4 hours: p= 0.21<br>6-8 versus 2-4 hours: p= 0.35<br>>8 versus 2-4 hours: p= 0.68	Barraquer et al (11)	2019	Spain
	No	PK: 8252 DSAEK: [illegible]	Retrospective national database	PK and DSAEK	Various	PK: 9 years DSAEK: 6 years	Survival	PK: Median= 9 hours, range= 0-33 hours, p= 0.05<br>DSAEK: Median= 10 hours, range= 0-26 hours, p= 0.1	Williams et al (24)	2015	Australia
	No	377	Retrospective cohort study	PK	Various	1 year	Failure	Total mean= 25.5 hr, Failure mean= 25.1 hr, HR= 0.99 (95% CI:0.98-1.02), p= 0.9	Yu et al (13)	2014	Germany
	No	11498	Retrospective national database	PK	Various	15 years	Survival	Mean survival:1 day: 12.22 years (95% CI: 11.55-12.88)<br>2 days: 12.24 years (95% CI: 11.66-12.82)<br>3 days: 12.17 years (95% CI: 11.69-12.65)<br>4 days: 12.08 years (95% CI: 11.43-12.73)<br>5 days:12.82 years (95% CI: 11.89-13.75)<br>6 days: 11.07 years (95% CI: 10.35-11.79)<br>7 days: 9.01 years (95% CI: 7.94-10.07)<br>>7 days: 10.17 years (95% CI: 9.08-11.26)	Williams et al (28)	2007	Australia
**Death to preservation time**	No	140	Retrospective cohort study	PK	Various	10 years	Survival	Rejection-free survival: ≤8 hours: n=72, median= 25.0 months (95% CI: 4.8-45.1)<br>9-14 hours: n= 48, median= 27.0 months (95% CI: 3.4-50.5)<br>≥15 hours: n=20, median= 36.0 months<br>p= 0.38<br>Overall survival: ≤8 hours: n=22<br>9-14 hours: n=20, median= 85.0 months (95% CI:30.9-139.0)<br>≥15 hours: n=6<br>p= 0.8	Kwon et al (8)	2020	Korea
	No	990	Prospective observational study	PK	Various	1 month	Failure	Primary failure: <6 hours= 1.3% (n=3), 6-12 hours= 3.5% (n= 4), ≥12 hours= 0 (n= 82), p= 0.13<br>Further information NA	Patel et al (14)	2017	New Zealand
	No	1629	Retrospective national database	PK	Various	1 year	Survival	5-hour intervals with no significant difference in survival rate, p= 0.47	Patel et al (9)	2011	New Zealand
	No	885	Retrospective case series	PK	Various	5 years	Survival	<5 hours= 82.8% n= 290, 5-<6.9 hours= 80.5% n=172<br>6.5-<9 hours 81% n= 226, ≥9 hours= 78.5% n=222<br>HR= 1.02 (95% CI: 0.97-1.08) (per +1 hour) , p= 0.42	Wagoner et al (15)	2010	Saudi Arabia
	No	99	Retrospective case series	PK	Various	2 years	Survival and failure	Clear grafts: mean age= 5.28 hours (SD= 2.8)<br>Unclear grafts: mean time= 4.45 hours (SD= 3.1)<br>Mean time for all grafts= 5.28 hours (SD= 2.8)<br>Further information NA	Chang et al (33)	1996	USA
**Enucleation to storage time**	No	3684	Retrospective national database	DSAEK	Various	8 years	Survival	0-3 hours: n= 2460, 4-6 hours: n= 535, 7-12 hours: n= 287, >12 hours: n= 402<br>Not an independent factor significantly affecting survival, p= 0.08	Williams et al (20)	2018	Australia
**Death to transplant time**	No	1040	Prospective cohort study	PK	Various	20 years	Survival	HR 1.01 (95% CI: 1.00-1.01), p= 0.25	Anshu et al (16)	2021	Singapore
	No	895	Retrospective cohort study	PK	Various	15 years	Survival	<12 versus 12-24 hours: p= 0.59<br>24-36 versus 12-24 hours: p= 0.71<br>>36 versus 12-24 hours: p= 0.72	Barraquer et al (11)	2019	Spain
	No	16650	Retrospective national database	PK	Various	15 years	Survival	Mean survival: >0 and ≤3 hours: 12.30 years (95% CI: 11.89-12.71)<br>>3 and ≤6 hours:12.77 years (95% CI: 12.24-13.29)<br>>6 and ≤9 hours: 11.15 years (95% CI: 10.61-11.68)<br>>9 and ≤12 hours:11.85 years (95% CI: 11.06-12.64)<br>>12 hours: 11.67 years (95% CI: 10.19-13.15)<br>Overall p=0.24	Williams et al (28)	2007	Australia
	Yes	210	Comparative retrospective nonrandomized clinical trial	PK	Various	2 years	Rejection	3 groups: fresh versus <7days versus >7 days storage<br>>7 days versus fresh: rejection: p<0.037, failure: p= 0.04<br>similar rejection in <7 days versus fresh	Simon et al (60)	2004	Austria
	No	539	Retrospective observational study	PK	Various	30 months	Failure	Failue with longer time: RR= 1.0 (95% CI:1.0-1.0), p= 0.90 as a continuous variable.<br>No significant association was found when analyzed as a categorical variable. Further information NA	Boisjoly et al (35)	1993	Canada
	No	63	Prospective case control	PK	Not specified	5 years	Survival	≤24 hours= 81%, >24 hours= 80.3%, not significant, Further information NA	Andersen and Ehlers (36)	1988	Denmark
**Storage to transplant time**	No	140	Retrospective cohort study	PK	Various	10 years	Survival	Rejection-free survival: ≤96 hours: n= 8, median= 12.0 months (95% CI: 0.9-23.0)<br>97–168 hours: n= 129, median= 33.0 months (95% CI:21.0–44.9)<br>169–240 hours: n= 3, median= 17.0 months<br>p= 0.78<br>Overall survival: ≤96 hours: n= 3<br>97–168 hours: n= 44, median= 85.0 months (95% CI: 59.7-110.2)<br>169–240 hours: n= 1 median= 21.0 months<br>p= 0.78	Kwon et al (8)	2020	Korea
	No	16746	Retrospective national database	PK	Various	29 years	Survival	Hypothermic medium: p= 0.001 (not an independent factor in multivariate analysis)	Williams et al (20)	2018	Australia
	No	1252	Registry study	PK and DSAEK	Various	2 years	Rejection and failure	≤29 days: n= 808, mean 20± 6 days, ≥29 days: n= 444, mean 38±7 days<br>PK: n= 417<br>Failure: p= 0.74<br>Rejection: p= 0.95<br>DSAEK: n= 834<br>Failure: p= 0.9<br>Rejection: p= 0.08	Madzak et al (18)	2018	Denmark
	No	1330	Multicenter, double-masked, randomized, noninferiority clinical trial	DSAEK	FED, ABK and PBK	3 years	Rejection	0-7 days n= 675 HR= 1<br>8-14 days n= 655 HR= 1.04 (95% CI: 0.47%-2.27%)<br>p= 0.91	Stulting et al (66)	2018	USA
	No	1330	Multicenter, double-masked, randomized, noninferiority clinical trial	DSAEK	FED, ABK and PBK	3 years	Survival	0-7 days= 95.3% (95% CI: 93.6%-96.9%)<br>8-14 days= 92.1% (95% CI: 89.9%-94.2%)<br>Conditional probability of failure after 1 month: 2.4% in 0-7 days versus 3.1% in 8-14 days	Rosenwasser et al (17)	2017	USA
	No	PK: 279 DSAEK: 1069	Retrospective national database	PK and DSAEK	Various	PK: 9 years DSAEK: 6 years	Survival	PK mean survival: <2 weeks: 4.25 years (95% CI: 3.74-4.75)<br>2-3 weeks: 4.36 years (95% CI: 3.95-4.76)<br>>3-4 weeks: 4.47 years (95% CI: 3.83-5.10)<br>p= 0.75<br>DSAEK mean survival: ≤5 days 3.97 years (95% CI: 3.70-4.23)<br>6-7 days 4.25 years (95% CI: 3.84-4.66)<br>>7 days 4.10 years (95% CI: 3.55-4.65)<br>p= 0.13	Williams et al (24)	2015	Australia
	No	377	Retrospective cohort study	PK	Various	1 year	Failure	Total mean storage= 19.26 days, Failure mean storage = 19.23 days, HR= 0.99 (95% CI:0.96–1.04), p= 0.97	Yu et al (13)	2014	Germany
	No	1629	Retrospective national database	PK	Various	1 year	Survival and failure	Survival= 12 days, SD= 4.3 days<br>Failure= 11.8 days, SD= 4.1 days, p= 0.54<br>Stratification into 5-day intervals (max 25 days), p= 0.39	Patel et al (9)	2011	New Zealand
	No	4142	Retrospective national database	PK	Various	10 years	Survival	Mean survival: 0 - 48 hours: 8.99 years (95% CI: 8.44-9.53)<br>49 - 96 hours: 9.10 years (95% CI: 8.19-10.01)<br>97 - 144 hours: 8.76 years (95% CI: 8.19-9.33)<br>≥145 hours: 8.18 years (95% CI: 7.39-8.97)<br>Overall p=0.45	Williams et al (28)	2007	Australia
	No	885	Retrospective case series	PK	Various	5 years	Survival	<175 hours= 81.9% n=235, 175-209 hours= 81.9% n=230<br>210-245 hours= 82.4% n= 219, >245 hours= 77.3% n= 226<br>HR= 1.01 (95% CI: 0.99, 1.03) p= 0.94	Wagoner et al (15)	2010	Saudi Arabia
	No	234	Retrospective case series	PK	Various	3 years	Survival	Optisol-GS storage time 7-8.99 days n= 73 (76.0%)<br>9-10.99 days n= 65 (85.5%)<br>11.0-14.5 days n= 16 (25.8%)	Wagoner et al (61)	2005	Saudi Arabia
**Preservation**	No	801	Retrospective cohort study	PK	Various	15 years	Survival	Preserved= 56%, Not preserved= 71%, HR>1, p= 0.1<br>Further information NA	Barraquer et al (11)	2019	Spain
**Storage media**	Yes	841	Retrospective case series	DMEK	FED	1 year	Rejection	Rejection significantly higher in cold culture n= 197 (23.4%) versus organ culture n= 644 (76.6%), p = 0.02	Hayashi et al (62)	2020	Germany
	PK: No DSAEK: No	PK: 24334 DSAEK: 4733	Retrospective national database	PK and DSAEK	Various	PK: 29 years DSAEK: 9 years	Survival	Organ culture versus hypothermic storage<br>PK: p= 0.003 (not an independent factor in multivariate analysis)<br>DSAEK: p= 0.24	Williams et al (20)	2018	Australia
	No	PK: 8125 DSAEK: 1790	Retrospective national database	PK and DSAEK	Various	PK: 13 years DSAEK: 6 years	Survival	PK mean survival:<br>Optisol medium: 8.85 years (95% CI: 8.42-8.88)<br>Organ culture medium: 4.49 years (95% CI: 4.30-4.68)<br>p= 0.35<br>DSAEK mean survival:<br>Optisol medium: 4.16 years (95% CI: 3.94-4.39)<br>Organ culture medium: 2.94 years (95% CI: 2.43-3.44)<br>p= 0.002 (not an independent factor in multivariate analysis)	Williams et al (24)	2015	Australia
	Yes	17090	Retrospective national database	PK	Various	15 years	Survival	Mean survival: Moist Pot:12.61 years (95% CI: 11.47-13.75)<br>M-K Medium: 12.38 years (95% CI: 11.99-12.77)<br>Superseded media: 11.00 years (95% CI: 10.61-11.40)<br>Organ culture: 9.65 years (95% CI: 8.03-11.27)<br>Optisol: 9.16 years (95% CI: 8.75-9.57)<br>Overall p=0.001	Williams et al (28)	2007	Australia
	No	210	Multi-center, randomized, prospective, double-masked clinical trial	PK	NA	1 year	Survival	DexSol: 95%, DexSol + hEGF (10 ng/ml) + human insulin (10 micrograms/ml) (ProCell): 97%, p= 0.90<br>Further information NA	Lass et al (19)	1994	USA
	No	539	Retrospective observational study	PK	Various	30 months	Failure	M-K medium compared to K-sol or Dexsol media: RR= 0.6 (95% CI:0.3-1.1), p= 0.12<br>Further information NA	Boisjoly et al (35)	1993	Canada
	Yes	1218	Retrospective cohort study	PK	Various	36 months	Survival	Organ culture n= 401, Moist chamber n= 132, M-K medium n= 340<br>Organ culture was most beneficial for survival (information NA), p=0.00002	Völker-Dieben et al (38)	1987	The Netherlands
**Pre-cut tissue**	DSAEK: No DMEK: Yes	DSAEK: 4736 DMEK: [illegible]	Retrospective national database	EK	Various	DSAEK: 10 years DMEK: 2-6	Survival	DSAEK:Pre-cut n= 1114, Surgeon-prepared n= 3622, p= 0.008 (significance not retained in final multi-variate analysis)<br>DMEK: Pre-cut n= 122 (2 years data), Surgeon-prepared n= 1128 (6 years data), p= 0.006	Williams et al (20)	2018	Australia
**Imported versus domestic cornea**	No	200	Retrospective cohort study	PK	Various	3 years	Failure	At 6-12 months domestic n=11, imported n=11<br>At 12-36 months domestic n=NA imported n=16, p>0.05<br>Further information NA	Singar-Ozdemir et al (69)	2018	Turkey
	No	146	Retrospective, nonrandomized review	PK	Various	18 months	Survival	Local 86%, Imported 88%, p= 0.2, Further information NA	Ababneh and Alomari (63)	2016	Jordan
**Donor epithelium**	No	232	Prospective, randomized clinical trial	PK	Various	4 years	Survival and rejection	Survival: epithelium-on= 91%, epithelium-off= 81%, p= 0.05<br>Rejection: epithelium-on= 2.7%, epithelium-off= 4.1%, p= 0.81<br>Further information NA	Stulting et al (64)	1988	USA

Abbreviatons: ABK= aphakic bullous keratopathy, CI = confidence interval, DALK= Deep Anterior Lamellar Keratoplasty, DMEK= Descemet Membrane Endothelial Keratoplasty, DSAEK= Descemet Stripping Automated Endothelial Keratoplasty, ECD= endothelial cell density, EK= endothelial keratoplasty, FED= Fuch's endothelial dystrophy, H-Y= histocompatibility Y, HLA= human leukocyte antigen, HR= Hazard ratio, Ig= immunoglobulin, KC= KC , M-K medium= McCarry Kaufman medium, n= number, NA= not available, OR= Odds ratio, p= probability, PBK= pseudophakic bullous keratopathy, PK= penetrating keratoplasty, RR= relative risk, NA = not available
